# Atypical Frequency Sweep Processing in Chinese Children With Reading Difficulties: Evidence From Magnetoencephalography

**DOI:** 10.3389/fpsyg.2020.01649

**Published:** 2020-08-11

**Authors:** Natalie Yu-Hsien Wang, Chun-Han Chiang, Hsiao-Lan Sharon Wang, Yu Tsao

**Affiliations:** ^1^Department of Audiology and Speech-Language Pathology, Asia University, Taichung, Taiwan; ^2^Department of Special Education, National Pingtung University, Pingtung, Taiwan; ^3^Department of Special Education, National Taiwan Normal University, Taipei, Taiwan; ^4^Research Center for Information Technology Innovation, Academia Sinica, Taipei, Taiwan

**Keywords:** reading difficulties, basic auditory processing, frequency sweep, magnetoencephalography, P1m

## Abstract

Chinese lexical tones determine word meaning and are crucial in reading development. Reduced tone awareness is widely reported in children with reading difficulties (RD). Lexical-tone processing requires sensitivity to frequency-modulated sound changes. The present study investigates whether reduced tone awareness in children with RD is reflected in basic auditory processing and the level at which the breakdown occurs. Magnetoencephalographic techniques and an oddball paradigm were used to elicit auditory-related neural responses. Five frequency sweep conditions were established to mirror the frequency fluctuation in Chinese lexical tones, including one standard (level) sweep and four deviant sweeps (fast-up, fast-down, slow-up, and slow-down). A total of 14 Chinese-speaking children aged 9–12 years with RD and 13 age-matched typically developing children were recruited. The participants completed a magnetoencephalographic data acquisition session, during which they watched a silent cartoon and the auditory stimuli were presented in a pseudorandomized order. The results revealed that the significant between-group difference was caused by differences in the level of auditory sensory processing, reflected by the P1m component elicited by the slow-up frequency sweep. This finding indicated that auditory sensory processing was affected by both the duration and the direction of a frequency sweep. Sensitivity to changes in duration and frequency is crucial for the processing of suprasegmental features. Therefore, this sensory deficit might be associated with difficulties discriminating two tones with an upward frequency contour in Chinese.

## Introduction

Reading development is associated with phonological awareness—the ability to identify and manipulate the components of speech sound (National Reading Panel, 2000; Kirby et al., 2003). Reduced phonological awareness has been widely observed among children with reading difficulties (RD) across language systems (Ziegler and Goswami, 2005; Goswami et al., 2011). Therefore, phonological processing deficits are considered the root cause of RD. A possible explanation for this deficit is that difficulties in basic auditory processing lead to inaccurate speech sound identification and mismatched representations, which constrain phonological processing (Tallal and Gaab, 2006; Pasquini et al., 2007). Basic auditory processing difficulties are likely to affect various domains of speech sound processing, such as detecting the fluctuations of sound intensity over time (amplitude-modulated speech signal; AM), the fluctuations of sound frequency over time (frequency-modulated speech signal; FM), and changes in phoneme duration. FM sound processing has received particular attention in research on basic auditory processing deficits in Chinese-speaking children with RD because of the close association between Chinese lexical tones and FM sound (Meng et al., 2005; Wang et al., 2019). Despite that behavioral evidence has reported reduced performance on the FM sound processing (Wang et al., 2019), it remains unclear how the deficit is reflected in neural response. This study aims to identify the neural processes associated with the atypical auditory processing of FM signals in Chinese-speaking children with RD.

### Lexical Awareness, Frequency Modulation, and Reading Ability

Chinese has four lexical-tone variations. All Chinese characters are monosyllabic, and the meaning of each character is defined by the lexical tone accompanying the syllable. The lexical tone is a suprasegmental feature that carries both prosodic and semantic information. For instance, associating the syllable/*ba*/with tone 1 signifies *eight* [八]; however, the meaning changes into *pull* [拔], *target* [爸], and *father* [爸] when the same syllable is associated with tones 2, 3, and 4, respectively. As isolated syllables, the patterns of the four tones are categorized by their fundamental frequency (F0) and pitch contour into high-level, high-rising, low-falling-rising, and high-falling (Xu et al., 2002). Because of the function of lexical tone, being aware of and familiar with the variation of lexical tones is necessary for Chinese character reading and learning new vocabulary (Tong et al., 2015).

Sensitivity to lexical tones is developmental and unique to tonal language speakers. Chinese-speaking children can accurately perceive variations in lexical tones at the age of approximately 1 year (Tsao, 2008, 2017). Mounting evidence demonstrates a correlation between lexical-tone awareness and reading-related abilities (McBride-Chang et al., 2008; Shu et al., 2008; Li and Ho, 2011). For example, Chinese character recognition (Wang et al., 2017), and reading comprehension (Choi et al., 2017; Liu and Tsao, 2017) are strongly correlated with performance on lexical-tone awareness tasks among children. A recent study investigating 90 children with RD and a matched number of their typically developing peers revealed a later developmental ceiling of lexical-tone awareness in children with RD (Wang et al., 2017). In a study by Wang et al. (2017), primary school children with and without RD from three different age groups (second-, fourth-, and sixth-grader students) were recruited to investigate lexical-tone awareness, verbal short-term memory, rapid automatic naming, and phonological awareness, which involves phoneme discrimination. Children with RD exhibited lower performance than their typically developing peers in all tasks. Moreover, in the fourth grade, the maturation of lexical-tone awareness was observed in typically developing children but not in children with RD. Therefore, lexical-tone awareness was considered a sensitive indicator of RD throughout primary school years.

### Basic Auditory Processing in RD

Evidence from alphabetic language speakers has indicated that children and adults with RD have difficulty detecting longer time-scale patterns of prosody-related sound features, such as duration, rhythm, AM, FM, and amplitude envelope rise time (Baldeweg et al., 1999; Goswami et al., 2002; Richardson et al., 2004; Pasquini et al., 2007). For instance, Richardson et al. (2004) assessed the performance of children with and without RD in discriminating linear rise and fall time envelopes, detecting rise time onset, duration discrimination, intensity detection, and rapid pitch discrimination. These tasks required amplitude envelope rise time processing, which was reported to account for the significant variance in phonological processing performances. Pasquini et al. (2007) reported similar findings in adults with RD; reading attainment was predicted by their performance on rise time perception and temporal order judgment. Strategies to compensate for these hearing deficits were not observed, as academic high achievers with reading problems performed worse than their peers on AM and FM detection tasks (Witton et al., 2002).

Basic auditory processing deficits are a probable contributor to reduced lexical-tone awareness because phonological deficits in RD are a cross-linguistic phenomenon. However, few studies have investigated basic auditory processing in the Chinese-speaking population with RD. Based on the hypothesis that lexical-tone processing involves sensitivity to spectrotemporal acoustic cues, Wang et al. (2019) investigated the relationship between basic auditory processing, lexical-tone awareness, and Chinese word recognition. The two auditory processing tasks required the participants to discriminate two sound sequences with frequency variations and identify the direction of frequency sweeps, which mimic the frequency changes in Chinese lexical tones. Their findings revealed that the ability to identify frequency sweep direction correlated strongly with both lexical-tone awareness and Chinese character recognition. Moreover, frequency sweep direction identification accounted for 11% and 19% of the variance in Chinese character recognition and lexical-tone awareness, respectively, indicating that basic auditory processing contributes to linguistic processing in Chinese-speaking children with RD.

### Neural Auditory and Processing Responses to Lexical Tone

Few event-related potentials (ERP) are associated with the sound discrimination used to identify the sensitivity of phonological awareness and the corresponding basic auditory processing. Mismatch negativity (MMN) and late discriminative negativity (LDN) are the most widely used components for measuring pre-attentive detection of an odd stimulus in a sound sequence. MMN peaks at approximately 150–250 ms from change onset, whereas LDN peaks at 300–600 ms. The two components are typically investigated using an oddball paradigm, in which one standard sound occurs regularly, and several deviant sounds with one or more different features appear irregularly in a sound sequence. ERP studies have reported that children with RD demonstrated atypical MMN or LDN patterns in perceiving both speech and basic auditory stimuli (Alonso-Búa et al., 2006; Huttunen et al., 2007; Neuhoff et al., 2012). Alonso-Búa et al. (2006) reported longer MMN latency in children with RD during the processing of speech stimuli (syllables). Atypical LDN, lower amplitude, and reduced latency were observed during the processing of both speech and non-speech (complex tones) stimuli. However, Hämäläinen et al. (2013) reported that kindergarten children with a high risk of RD exhibited stronger N250 responses, resembling MMN, to both speech stimuli (pseudowords), and non-speech stimuli (sounds with formant and frequency mimicking real phonemes) compared with a control group. Evidence regarding MMN and LDN is inconsistent across studies, which may be because of the task designs and the profiles of the populations involved. However, this discrepancy indicates that atypical auditory and speech processing occurs at the neural level.

P300 (P3) is another ERP component that is reportedly associated with RD. P3 is a late positive component that occurs at approximately 300 ms after the stimulus onset. It is linked to the process of context updating, working memory, and decision-making. Similar to MMN and LDN, P3 is usually elicited by using an oddball paradigm, which reflects higher-cognitive functions that may influence the speed of auditory processing (Polich, 1987). Studies have indicated that P3 may be an effective indicator of central auditory system integrity and auditory attention functioning (Mendonça et al., 2013). Maciejewska et al. (2013) reported prolonged P3 latencies in children with RD during a non-speech auditory processing task. Maciejewska et al. (2013) highlighted that P3 latency was modulated by age in typically developing children. Older children should exhibit a shorter latency; however, the age effect was absent in children with RD. This finding suggests that maturation of non-speech auditory processing may be delayed, which contributes neurological evidence to the behavioral-based account presented by Wang et al. (2017). Furthermore, Papagiannopoulou and Lagopoulos (2017) reported a reduced P3 amplitude and localization response in children with RD, suggesting a delayed processing speed, and a diminished processing capacity.

The phonological deficit hypothesis for RD has been proven to be universal, and processing of FM sound and lexical-tone awareness are closely associated; therefore, Chinese-speaking children with and without RD may exhibit neural-level differences in basic auditory processing. Little research has been conducted to verify this hypothesis. Meng et al. (2005) clarified neural-level differences that may reflect the difficulties in lexical-tone processing. They reported a reduced performance in tone frequency and temporal discrimination tests in children with RD. Furthermore, atypical ERPs were elicited by a steady pure tone discrimination task. Although the results were not statistically significant, children with RD demonstrated lower MMN than their typically developing peers. However, steady pure tone stimuli do not optimally reflect the dynamic nature of Chinese lexical-tone variations, because only tone 1 is flat and the other tones are composed of rising or falling frequency changes. Furthermore, the MMN component alone does not necessarily reflect the level of auditory processing breakdown. Therefore, further research is needed to explore auditory processing difficulties among Chinese-speaking children with RD.

### Aims of the Study

Few behavioral studies have investigated the association between lexical-tone processing and basic auditory processing. Therefore, this study focused on verifying whether the difference in basic auditory processing between children with and without RD occurred at the neural level. A magnetoencephalography (MEG) technique was used, because this approach provides a spatial resolution more favorable than that of electroencephalography (EEG). A frequency sweep was employed as the auditory stimuli; this created a non-linguistic condition that resembled the characteristics of Chinese lexical tones.

## Methodology

### Participants

In Taiwan, the majority of children with RD are diagnosed after the third grade; a potential diagnosis is made for those who demonstrate relatively severe reading impairments at an early stage of primary school education. Third- and fourth-grader students with normal hearing were recruited for this study, including 13 children with RD and 14 of their typically developing peers (controls). The children with RD (mean age = 114.8 months; *SD* = 11.82; 11 boys and 2 girls) were identified from the database of the Special Education Division, Department of Education of Taipei City Government. The main inclusion criteria for the children with RD were significant Chinese character decoding failure and a demonstrated delay > 1 year compared with the controls. The controls were age-matched (mean age = 112.57; *SD* = 7.2; 10 boys and 4 girls) children from local primary schools who volunteered for the study. The sex ratio did not differ between groups [*X*^2^(1) = 0.68, *p* = 0.65]. According to reports from parents and schoolteachers, the controls had no learning difficulties and no neurological or psychiatric disorders. Before the experiment, participants and their parents were debriefed, and informed consent was obtained. The study was approved by the Research Ethics Office of National Taiwan University (Application No. 201310EM016).

All children were assessed using the Graded Chinese Character Recognition Test (Huang, 2001) and the Abbreviated Wechsler Intelligence Scale for children, to evaluate their character recognition performance and ensure a normal intelligence quotient (IQ). As demonstrated in Table 1, both groups had a full-scale IQ of >80, and children with RD exhibited results 1.5 standard deviations lower than the controls in Chinese character recognition.

**TABLE 1:** Average scores of participants’ age, IQ, and Chinese character recognition.

	**CA (*N* = 14)**	**RD (*N* = 15)**	***t***
Age (months)	109.79 (8.54)	114.20 (11.93)	–1.138
IQ (full scale)	110.39 (10.56)	92.59 (11.89)	4.249**
Chinese character recognition	85.71 (26.17)	49.53 (23.60)	3.914*

Standard deviations are presented in the bracket. *p < 0.01; **p < 0.001.

### Stimuli and Procedure for the MEG Experiment

Four sound sequences for FM sweep were used as auditory stimuli. Each sound sequence was composed of the repetition of a standard and four FM sweep deviant variations (fast-up, fast-down, slow-up, and slow-down). The standard FM sweep was created with a combination of 180- and 600-Hz stimuli; the duration of the sound was 240 ms. The directions of the deviant FM sweeps were either upward or downward, and the conditions of the speed of frequency change were either 80 and 160 ms. The fast-up FM sweep involved a frequency increase from 180 to 270 Hz over 80 ms, whereas in the fast-down FM sweep the frequency decreased from 270 to 180 Hz. The slow-up and slow-down FM sweeps involved a frequency increase from 600 to 900 Hz and a frequency drop from 900 to 600 Hz, respectively, over 160 ms.

The order in which the FM sweeps were presented in the sound sequences was pseudorandomized. Each sound sequence contained 360 deviant FM sweeps (90 per deviant condition) and 840 standard sounds. A total of 20 standard sounds were placed at the beginning of a sound sequence, and the other standard sounds were inserted between the deviant FM sweeps to ensure that the spacing was appropriate but unpredictable. The overall duration of a sound sequence was approximately 5 min. The visual stimulus was a silent cartoon divided into four sections, equal in length to the sound sequence.

The MEG experiment adopted an oddball paradigm with a block design. During MEG data acquisition, the children were presented with a silent cartoon along with the FM sweep sequence. The children were told that no response was needed. The experiment contained four blocks, and the overall time of data acquisition was 25–30 min.

### MEG Data Acquisition

Data acquisition was performed using a whole-head 306 sensor MEG device (Elekta Neuromag, Helsinki, Finland) with 102 arrays, containing one magnetometer and two orthogonal, planar gradiometers. To monitor the head location in relation to the MEG sensors, four Head Position Indicator coils with a 293–321-Hz sinusoidal current were attached to the scalp. The sampling rate was 1000 Hz, and a band-pass filter of 1–40 Hz was employed. Eye movements during the acquisition were recorded with two electrodes attached above and to the side of the left eye.

### MEG Data Analysis

The data were preprocessed using single subspace separation (Taulu et al., 2005) in the MaxFilter program (Elekta Neuromag) to reduce the strength of external noise. Head movement compensation (200 ms) was also performed using the MaxFilter program to transform the head origin to the same position for each participant.

The data were exported into BESA Research 6.0 (BESA GmbH, Gräfelfing, Germany) for averaging and dipole-source localization. A segmentation process was used to extract the time sequences between -200 and 800 ms for preprocessing with a 1-Hz high-pass filter and a 40-Hz low-pass filter. BESA Statistics was used to perform permutation tests—a non-parametric statistic method for channel and time-point clustering—to examine between-group and within-subject differences. *P*-values were calculated to assess statistical significance. The permutation testing of 1000 permutations was significant at alpha = 0.05, and the channel neighbor distance was 4 cm (for permutation testing, see Maris and Oostenveld, 2007). The equivalent current dipole models and CLARA source localization model in BESA Research 6.0 were used to investigate the brain regions responsible for the group differences.

## Results

### Effects of FM Sweeps on Event-Related Fields

**FIGURE 1:**
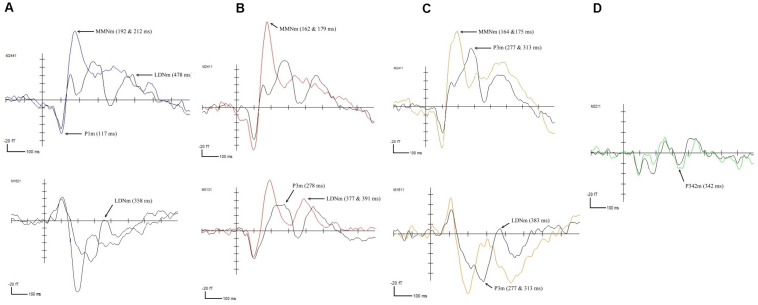
ERFs elicited in the control participants by **(A)** the fast-up sweep, **(B)** the fast-down sweep, **(C)** the slow-up sweep, and **(D)** the slow-down sweep.

The results of the permutation tests revealed that the four deviant FM sweep conditions elicited Event-Related Fields (ERFs) that were significantly different from the standard condition. However, the effects were reflected differently in the control and RD groups. In the control group, the fast-up sweep elicited deviant waveforms at five different time points, including a positive peak at 117 ms (*p* < 0.05) and negative peaks at 192 (*p* < 0.001), 212 (*p* < 0.0001), 358 (*p* < 0.05), and 478 ms (*p* < 0.01), as illustrated in Figure 1A. The positive peak, observed in the right anterior and left posterior temporal areas, corresponded to the P1m component. The negative change in the left middle temporal and right anterior temporal area waveforms resembled the MMNm component (192 and 212 ms) and the LDNm component (478 ms). The fast-down sweep elicited a negative change in the waveform, corresponding to the MMNm component at 162 (*p* < 0.0001) and 179 ms (*p* < 0.0001) and corresponding to the LDNm component at 377 (*p* < 0.05) and 391 ms (*p* < 0.01) in the bilateral temporal lobes, as illustrated in Figure 1B. Furthermore, the P3m component was observed at 278 ms (*p* < 0.01). The slow-up sweep, illustrated in Figure 1C, induced negative waveforms associated with the MMNm component at 164 (*p* < 0.01) and 175 ms (*p* < 0.01) and associated with the LDNm component at 383 ms (*p* < 0.01), in the right temporal area. A positive peak that corresponded to the P3m component was identified at 277 ms (*p* < 0.0001). For the slow-down sweep condition, illustrated Figure 1D, only the P3m component at 342 ms (*p* < 0.01) was observed in the left posterior temporal lobe.

**FIGURE 2:**
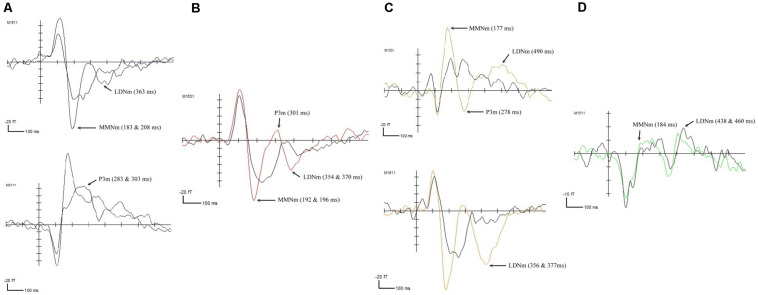
ERFs elicited in the children with RD by **(A)** the fast-up sweep, **(B)** the fast-down sweep, **(C)** the slow-up sweep, and **(D)** the slow-down sweep.

In the RD group, the fast-up sweep, illustrated in Figure 2A, triggered negative waveform changes that corresponded to the MMNm component at 283 ms (*p* < 0.01) and the LDNm component at 363 ms (*p* < 0.0001) in the bilateral temporal regions. The P3m component, at 303 ms (*p* < 0.01), was only observed in the left temporal area. The fast-down sweep elicited the MMNm component at 192 (*p* < 0.001) and 196 ms (*p* < 0.01) in the bilateral temporal lobes, as illustrated in Figure 2B. Negative changes in the waveforms were also observed at 354 (*p* < 0.001) and 370 ms (*p* < 0.01), which were associated with the LDNm component, in the right posterior frontal area and the left temporal area. A positive peak was observed at 301 ms (*p* < 0.01) in the right temporal area. Similarly, the slow-up sweep, illustrated in Figure 2C, triggered negative waveform changes associated with the MMNm component at 177 ms (*p* < 0.0001) and the LDNm component at 490 ms (*p* < 0.01) in the bilateral temporal lobes. A positive waveform peak at 278 ms, associated with the P3m component, was also observed in this area. For the slow-down sweep, the MMNm component at 184 ms (*p* < 0.0001), and LDNm component at 460 ms (*p* < 0.0001) were observed in the left and the right temporal lobes, respectively, as illustrated in Figure 2D.

### Group Effect on ERFs

**FIGURE 3:**
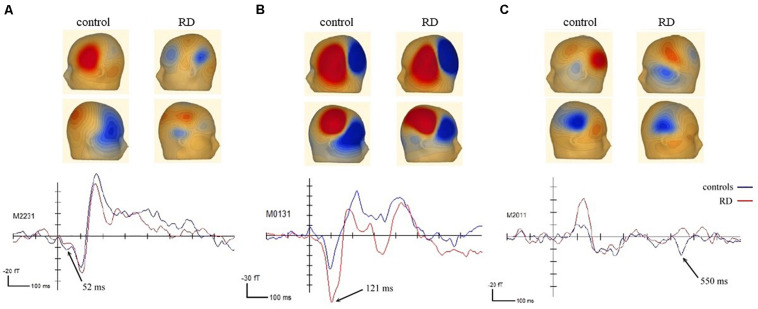
Between-group differences in ERF observed for three different time points and sources: ERF with peak time at **(A)** 52 ms in the left inferior frontal gyrus, **(B)** 121 ms in the left frontal region, and **(C)** 550 ms in the left middle frontal gyrus.

A permutation test demonstrated between-group differences in the waveform changes. Significant between-group differences were reported in three of the four FM sweep conditions. First, a significantly different cluster in the central frontal field map was identified in the fast-up sweep condition. As illustrated in Figure 3A, the difference occurred at 30–95 ms (*p* = 0.041) with a peak time at 52 ms. The equivalent current dipole models and CLARA source localization model, with 49.59% residual variance, further revealed that the ERF source was in the left inferior frontal gyrus.

Second, a between-group difference was observed in the slow-up sweep condition between 105 and 140 ms (*p* = 0.035), as displayed in Figure 3B. The distribution of the upward field peaked at 121 ms in the left frontal area. However, source localization models (residual variance = 19.33%) further revealed differences in the left inferior frontal gyrus (blue dipole) and the postcentral gyrus in the right parietal lobe (red dipole). Third, a between-group difference in the slow-down condition was revealed. A downward current was observed during 535–570 ms with a peak at 550 ms (*p* = 0.034) in the left occipital area, as displayed in Figure 3C. The source localization results suggested that the significant clusters were close to the left middle frontal gyrus (blue dipole) and the cingulate gyrus in the right hemisphere (red dipole). The residual variance was 27.63%.

## Discussion

This study explored the differences in basic auditory processing between Chinese-speaking children with and without RD. The results demonstrated that the oddball paradigm elicited three auditory components (i.e., MMNm, LDNm, and P3m) commonly associated with detecting sound changes in both groups. However, the effects of sound changes were inconsistent across the frequency sweep conditions and groups. For the MMNm component, the control group exhibited a larger amplitude than the children with RD in the fast-up, fast-down, and slow-up conditions; the MMNm component was not observed in the slow-down frequency sweep condition in the control group, and the RD group demonstrated a relatively low amplitude. The patterns of the LDNm component mirrored the MMNm in the larger amplitude observed in the control group in all frequency sweep conditions except the slow-down condition, which exhibited no effect in the control group and a low amplitude effect in the RD group. Although differences in neural response patterns may be observed, permutation tests failed to reveal group effects on these pre-attentive auditory components. However, the P3 component was less stable and was only observed in the fast-down and slow-up frequency sweep conditions. Although the delayed latency was observed for the fast-down frequency sweep in the children with RD, the evidence is inconclusive regarding deficits in auditory attention or central auditory system functioning. However, other cognitive deficits, including attention disorders, were exclusion criteria when recruiting the children with RD and thus the neural responses of attention-related components were not significantly different from those in typically developing children.

The findings of this study indicate that basic auditory processing deficits in children with RD may not be as strongly associated with pre-attentive auditory processing as originally expected. Although the MMN component identified in children with RD differed from that in the control group in terms of the amplitude and latency of ERP, the differences were not significant in phonological or basic auditory passive listening tasks. This accorded with findings reported by Meng et al. (2005). These combined findings suggest that the MMN component may not be an efficient indicator of basic auditory processing deficits in Chinese-speaking children with RD. The main difference between these two studies was the design of basic auditory processing stimuli. Meng et al. (2005) used steady pure tones of different frequencies, whereas the present study employed frequency sweeps that reflected the frequency fluctuation within Chinese lexical tones. Nonetheless, the findings were consistent, suggesting the pre-attentive auditory processing is not the major contributor to the basic auditory processing deficits observed in Chinese-speaking children with RD. Analysis of the LDNm component, which also reflects pre-attentive auditory processing, did not reveal significant differences between the groups, supporting the argument that the basic auditory processing deficits may not involve the pre-attentive level.

However, between-group differences were observed over three different time frames, namely, 30–95, 105–140, and 535–570 ms. Only the positive peaks at 52 and 121 ms were considered as auditory evoked fields. The findings were interpreted carefully because the two early peaks may reflect auditory sensory processing in the P1m component, which matures with age (Ponton et al., 2000). P1m deflection in newborns peaks at approximately 250 ms, but, as children age, it becomes smaller, and its latency decreases (Ponton et al., 2000). According to Paetau et al. (1995), the auditory evoked field of P1m peaks at approximately 50 ms in adults, whereas the peak occurs at approximately 100 ms in children aged 3 months to 12 years. Therefore, we considered that the peak observed at 121 ms resembled the P1m component in the children involved in this study. The P1m component has been associated with early auditory sensory processing in the central auditory system. This study demonstrated that the children with RD demonstrated weaker P1m response compared with their typically developing peers, suggesting potential auditory sensory processing deficits.

In the existing literature, the P1(m) component is often investigated along with the N2(m) component, which is a negative waveform that occurs around 200 ms, to reflect auditory sensory processing in the perception of basic auditory stimuli which employed mainly duration and frequency (Swink and Stuart, 2012). Sensitivity to sound duration and frequency are closely associated with the processing of suprasegmental features, such as stress, tone, and intonation. Chung and Bidelman (2016) suggested that healthy individuals with different levels of language proficiency (native and non-native adult speakers of English) demonstrated distinct waveform patterns during the processing of pseudowords with a legal English stress. This finding suggested that auditory sensory processing could be an indicator of phonological awareness at the suprasegmental level.

Studies on alphabetic-language-speaking children with RD or with a high risk of RD have demonstrated an altered P1(m) component in various basic auditory processing tasks (Lovio et al., 2010; Khan et al., 2011; Stefanics et al., 2011). For instance, Lovio et al. (2010) reported in their ERP study that children with a risk of RD demonstrated altered neural response to syllable duration. The appearance of a syllable in an oddball paradigm elicited different P1 responses in children at risk of RD and the control group. The children at risk of RD demonstrated overall smaller P1 amplitudes than the controls in the frontocentral regions. The amplitude envelop rise time is a critical acoustic property underlying syllable rate (Drullman, 2006), and thus, sensitivity to the rise time may reflect the ability to process speech (Goswami, 2011). Stefanics et al. (2011) determined that reduced sensitivity to rise time in children with RD was associated with atypical P1 responses in the frontocentral regions; furthermore, the effect of stimuli conditions (speed of rise time) engendered variations in the responses of children with RD, whereas this phenomenon was not observed in the control group. The children with RD had smaller P1 responses to slower rise time stimuli than for faster rise time stimuli. Stefanics et al. (2011) hypothesized that difficulties in processing were associated with an extended rise time. These findings partially accorded with the results of the present study.

In this study, the atypical P1m responses in the children with RD were also observed in the frontocentral regions, including the left inferior frontal gyrus and the postcentral gyrus in the right parietal lobe. However, the atypical responses in children with RD were unexpected, because the slow-up frequency sweep exhibited a between-group difference in the P1m component. Furthermore, the children with RD demonstrated a larger amplitude than the controls in the processing of slow-up frequency. These findings contribute cross-linguistic evidence that children with RD exhibit auditory sensory processing deficits. Concordant with the aforementioned findings among English-speaking children with RD (Stefanics et al., 2011), the atypical P1m responses in Chinese-speaking children were elicited by stimuli with a slower rise time (the slow-up frequency sweep). This finding indicates that auditory sensory processing deficits were particularly evident for relatively extended auditory stimuli. However, the lack of group differences in P1m response to processing the slow-down frequency sweep suggested that the frequency sweep direction and the overall duration of a stimulus affect the basic auditory processing at the sensory level. A behavioral study by Wang et al. (2019) adopted upward and downward frequency sweeps with relatively short durations, seven variations between 5 and 80 ms. Between-group differences in discrimination of frequency sweep directions were reported; however, the effects of overall duration and direction on discrimination accuracy were not discussed. Therefore, we cannot conclude whether the manipulation of speed and frequency sweep direction in the study by Wang et al. and the current study have shared patterns. Moreover, in this study, the group differences in neural responses were elicited with stimuli of 160 ms rather than 80 ms, leading to the question of whether frequency sweeps with longer durations reflect children’s ability to discriminate frequency sweep direction more accurately. Further research combining behavioral and neurological evaluation is needed to clarify this aspect.

The findings contribute to the relevant literature on basic auditory processing in Chinese-speaking children and demonstrate that the reduced sensitivity to non-speech sounds has neurological origins. Furthermore, difficulty in distinguishing tone 2 from tone 3 is a commonly reported characteristic of Chinese-speaking children with RD, which may also be related to the phenomena observed in this study. Tone 3 resembles tone 2 in its rising contour; therefore, distinguishing the tones requires detection of the timing of turning point in tone 3 (Shen and Lin, 1991). Moreover, the overall durations of tone 2 (417.7 ms), and tone 3 (484 ms) are relatively longer than those of tones 1 (416.2 ms) and 4 (307.8 ms) in monosyllabic conditions (Yang et al., 2017). This discrimination is more challenging, even for typically developing children. Wong et al. (2005) reported that typically developing preschoolers perceived tone 1, tone 2, and tone 4 more accurately than tone 3, with accuracies of 90, 87, and 89%, respectively; the accuracy for identifying tone 3 was only 70%. Tone 3 was most frequently misidentified as tone 2. The group difference in neural responses was caused by the frequency sweep condition that resembled the relative duration and the sweep direction of the two easily confused tones. However, the present experimental design does not allow us to verify whether the P1m responses were directly associated with children’s accuracy in discriminating tones 2 and 3. Future research is needed to provide a deeper understanding of neural responses and children’s lexical-tone awareness and, specifically, whether the P1m component is particularly sensitive to the difficulty in tone 2 and tone 3 discrimination.

In conclusion, this study investigated whether Chinese-speaking children with RD demonstrated basic auditory processing and at what level the auditory processing deficits occurred. The findings of this study provide evidence that accords with studies that have reported basic auditory processing deficits in children with RD. Moreover, the major contribution of this study is the evidence that basic auditory processing deficits may result from disrupted auditory sensory processing rather than pre-attentive auditory processing, which has been a focus of investigation in numerous studies. Furthermore, the atypical auditory sensory processing was particularly evident when the frequency sweep direction was upward and the overall duration was longer. Auditory sensory processing difficulties may be tuned to the characteristics of auditory stimuli in children with RD. The interaction between stimuli duration and frequency sweep direction requires further research, verifying the effect on auditory sensory processing and clarifying the association with Chinese lexical-tone processing.

## Data Availability Statement

The datasets generated for this study are available on request to the corresponding author.

## Ethics Statement

The studies involving human participants were reviewed and approved by Research Ethics Office of National Taiwan University. Written informed consent to participate in this study was provided by the participants’ legal guardian/next of kin.

## Author Contributions

NW was responsible for data analysis and writing up the manuscript. C-HC was responsible for data collection and analysis. NW was the PI of this research project and supports the writing up of the manuscript. YT was the co-PI of this research and responsible for creating the auditory stimuli used for the experiment. All authors contributed to the article and approved the submitted version.

## Conflict of Interest

The authors declare that the research was conducted in the absence of any commercial or financial relationships that could be construed as a potential conflict of interest.
